# Cardiac and Hepatic T2*-Weighted Magnetic Resonance Imaging in Transfusion Dependent Hemoglobinopathy in North West of Iran

**Published:** 2015-12-10

**Authors:** N Valizadeh, V Alinejad, S Hejazi, M Noroozi, A Hashemi, B Rahimi, Sh Nateghi

**Affiliations:** 1 Assistant professor of Hematology/Medical Oncology, Urmia University of Medical Sciences, Urmia, Iran; 2 MSc of of Biostatistics, Patient Safety Research Center, Urmia University of Medical Sciences, Urmia, Iran; 3 Assistant professor of Pediatric Hematology/Oncology, Urmia University of Medical Sciences, Urmia, Iran; 4 Assistant professor of Pediatric Hematology/Oncology, Urmia University of Medical Sciences, Urmia, Iran; 5 Assistant professor of Gastroenterology, Urmia University of Medical Sciences, Urmia, Iran; 6 Assistant professor of Cardiology, Urmia University of Medical Sciences, Urmia, Iran; 7 General physician, Urmia University of Medical Sciences, Urmia, Iran

**Keywords:** Hemoglobinopathy, Heart, Iron, Liver, MRI

## Abstract

**Background:**

Iron overload is the main transfusion related side effects in patients with transfusion dependent hemoglobinopathies. Severe iron deposition in tissues leads to organ dysfunction. Many organs can be affected such as heart, liver, and endocrine organs. Cardiac failure and liver fibrosis are the consequent of Iron overload in transfusion dependent hemoglobinopathy. Magnetic Resonance Imaging (MRI) is a safe, noninvasive, and accurate method for the assessment of iron deposition in different tissues. This study assessed iron levels in liver and heart of the patients with transfusion dependent hemoglobinopathies.

**Materials and Methods:**

The studied population consisted of 12 patients (7 male and 5 female) with transfusion dependent hemoglobinopathies, aged between 10-18 years old. Then, Cardiac and liver T2*- weighted magnetic resonance imaging (MRI) were obtained.

**Results:**

In current study, 1patient (8.33%) had severe, 2 patients (16.66%) had moderate and 2(16.66%) had mild cardiac iron deposition. Out of 12 patients, 1 had severe iron deposition in liver (8.33%), 5(41.66%) and 4(33.33%) had moderate and mild hepatic iron deposition, respectively. Differences between Hepatic and cardiac iron levels were not significant between males and females (p>0.05).

**Conclusion:**

Since cardiac and liver iron levels were higher than normal in most of the study group, checking ferritin level and liver function test and also echocardiography in shorter intervals (each 3 months) in involved group is suggested instead of checking routinely in 6 month intervals in patients with transfusion dependent hemoglobinopathies.

## Introduction

Blood transfusion has increased the overall survival and quality of life in transfusion dependent hemoglobinopathies; but can lead to iron deposition and tissue damage especially in heart, liver, and endocrine glands. Cardiac iron deposition and related cardiomyopathy is the main cause of mortality in transfusion dependent thalassemia. Although Iron chelation therapy improved survival of patients with transfusion dependent hemoglobinopathies, it has own side effects and cannot be available permanently in some developing countries due to socioeconomic factors. Myocardial iron deposition may be independent from other tissues involvement and heart can be spared despite heavy iron deposition in other organs (1-3).

For diagnosis and treatment of iron overload in various body tissues, an accurate and safe method is needed.

Since liver is the first organ for iron storage in patients with transfusion dependent hemoglobinopathy and secondary hemochromatosis, thus liver iron content can be an indicator for total body iron storages.

Magnetic resonance imaging (MRI) had been a safe and accurate method for assessment of liver iron concentration (LIC) in the past decades in patients with transfusion dependent anemias and recently it was widely accepted for monitoring of iron chelation therapy (4-7).

MRI evaluation of cardiac iron level estimates cardiac risks and guides for iron chelation therapy.Introduction of cardiacMRI T2* view in the early 2000s impact on patients care as well as diagnosis of iron induced cardiomyopathy (8). In this study, Iron level in liver and heart of patients with transfusion dependent hemoglobinopathy was assessed. 

## Materials and Methods:

In this cross –sectional study, 12 children (7 male and 5 female) with transfusion dependent thalassemia treated conventionally, aged between 10-18 years, were enrolled and evaluated for cardiac and liver T2*-weighted magnetic resonance imaging (MRI). Inclusion criteria were any male or female with transfusion dependent hemoglobinopathy (including thalassemia major and intermedia) and exclusion criteria was any contraindication to MRI scans.

## Results

In the study group, 1patient(8.33%)had severe cardiac iron overload, 2 patients(16.66%)had moderate iron deposition in heart and 2 of them(16.66%)had mild cardiac iron deposition. In 7 cases (58.33%) cardiac iron level was normal (Table I).

 Out of 12 patients, 1 (8.33%) had severe iron deposition in liver and 5 (41.66%) and 4(33.33%) had moderate and mild iron deposition in their liver. In 2(16.66%) of them hepatic iron levels were normal (Table II).

Hepatic and cardiac sidrosis were not significant between males and females. In this study also did not find any correlation between cardiac and liver sidrosis.

There was not any statistical significant correlation between heart and liver iron overload in the study group (P-value =0.177).

Significant difference did not find between heart iron overload and sex of patents with Chi-squared test. (P-value=0.876)

There was not any significant difference between liver iron overload and sex of patents with Chi-squared test. (P-value=0.53)

**Table I:** Cardiac Iron Deposition

**Cardiac iron deposition**	**Frequency**	**Percent**
Severe	1	8.33
Moderate	2	16.66
Mild	2	16.66
Normal	7	58.33
Total	12	100

**Table II:** Hepatic Iron Deposition

Liver iron deposition	Frequency	Percent
Severe	1	8.33
Moderate	5	41.66
Mild	4	33.33
Normal	2	16.66
Total	12	100.0

## Discussion

Candini G, et al assessed cardiac and he patic iron overload in thalassemic patients by MRI and confirmed the usefulness of this method for monitoring the iron overload in patients with thalassemia (9).

Christoforidis, et al studied iron accumulation in liver, myocardium, and pituitary with MRI in 30 young thalassemic patients. They did not find statistical significant correlation between mean ferritin levels versus liver, pituitary, and cardiac MRI values (10).

Christoforidis et al, conducted a prospective study with serial MRI scans in young patients with beta-thalassemia major and compared between different chelation regimens and concluded that Liver MRI was better correlated with serum ferritin concentrations than myocardial MRI. Liver MRI values were highly correlated with LICs derived from percutaneous liver biopsy; whereas, myocardial MRI values did not correlate at all with measurements derived from echocardiography. Regarding iron chelation treatment, patients receiving combined therapy with deferiprone and deferoxamine (DFO) significantly reduced myocardial iron overload during the 4-yr study period, whilst patients in monotherapy with DFO showed a significant increase in LIC (11).

Although feriitin level is accepted widely for monitoring iron overload in patients with primary hemochromatosis, studies show that ferritinemia is not accurate than other methods such as direct measurement of iron in the liver and Magnetic Resonance Imaging (MRI).

Mazza P, et al compared magnetic resonance imaging, serum ferritin, and iron content of the liver for assessment of iron overload in patients with thalassemia and concluded that serum ferritin levels had a tendency to be significantly correlated with the status of hemochromatosis in thalassemia patients; however , the discrepancies reported in several patients and the scarce or total lack of correlation with MRI recommend using other procedures to iron overload to guide physicians about iron chelation therapy(12).

Pepe A, et al performed a multicenter prospective study for comparison of combined deferiprone and deferoxamine therapy against deferiprone or deferoxamine monotherapy on cardiac and hepatic iron and ejection fraction in thalassemia major patients and concluded that the combined deferiprone and deferoxamine( DFP+DFO) regimen was more effective in removing cardiac iron than DFO and was superior in clearing hepatic iron than either DFO or DFPmonotherapy but combination therapy had not an additional effect on heart function over DFP(13).

Iron chelators are used in patients with transfusion dependent hemoglobinopathies to remove excess iron and prevent from iron induced organ damages. Three chelators are available and commonly used in worldwide including deferoxamine, deferasirox, and deferiprone. For choosing each of them several factors including the cost of them, severity of hyperferritinemia, administration schedule, and side effects of them should be considered (14).

Combination therapy with two chelators is more effective in removing iron excess without compromising safety or compliance. The use of MRI-based technology also holds promise for wider application of non-invasive assessment of cardiac iron in the management of patients with thalassemia (15).

Karimi M, et al studied the results of hepatic MRI evaluation and comparison of liver signal intensities in the patients and the control group and showed that 4 suffered from (14.2%) mild, 7 (25.0%) moderate and 17 (60.8%) severe hemochromatosis(16).

In the study, 1(8.33%) had severe iron deposition in liver and 5 (41.66%) and 4(33.33%) of them had moderate and mild iron deposition in their livers which showed severe liver hemochromatosis. 

## Conclusion

Since cardiac and liver iron levels were higher than normal in most of the study group, checking ferritin level and liver function test and also echocardiography in shorter intervals (each 3 months) in involved group is suggested instead of checking routinely in 6 month intervals in patients with transfusion dependent hemoglobinopathies. 
